# Paraneoplastic Limbic Encephalitis in a Patient With Thymoma

**DOI:** 10.7759/cureus.84156

**Published:** 2025-05-15

**Authors:** Abdullah Alwohaibi, Wajd Althakfi, Hatoon Bakhribah

**Affiliations:** 1 Department of Medical Oncology, Comprehensive Cancer Center, King Fahad Medical City, Riyadh, SAU; 2 Histopathology Unit, Department of Pathology, King Saud University, Riyadh, SAU; 3 Department of Pathology, King Fahad Medical City, Riyadh, SAU

**Keywords:** caspr2 antibodies, castleman disease, contactin-associated protein-like 2, leucine-rich glioma-inactivated 1 (lgi1) antibody, lgi1 antibody autoimmune encephalitis, malignant thymoma, neuromyotonia, paraneoplastic limbic encephalitis (ple), paraneoplastic neurological syndromes, thymic malignancies

## Abstract

Paraneoplastic limbic encephalitis is a rare neurological disorder that is classically associated with small cell lung cancer, it usually presents with seizures, confusion, behavioral changes and cognitive impairments. It has been associated with a number of onconeural antibodies. Its association with other malignancies is less recognized. We present a case of a young male with a diagnosis of thymoma who developed limbic encephalitis after the diagnosis and the initial treatment of his malignancy. The patient showed clinical improvement after receiving radiotherapy to the primary tumor.

## Introduction

Paraneoplastic limbic encephalitis (PLE) is a rare paraneoplastic neurological disorder that has been reported with various malignancies, usually preceding the cancer diagnosis, but it can occurs at different stages of the disease. It typically presents with acute to subacute symptoms of memory impairment, cognitive dysfunction, behavioral changes, hallucinations, confusion and seizures [1]. Small cell lung cancer (SCLC) is the most common malignancy associated with PLE [2]. It is mediated by autoimmune antibodies directed to different neuronal antigens that can often be detected in serum or cerebrospinal fluid (CSF). Diagnosis is usually challenging, the presence of the typical neurological symptoms along with detection of auto-antibodies suffices for diagnosis. In the absence of auto-antibodies, a diagnosis can be made if typical findings in brain imaging and CSF examination are found [3]. PLE is managed by treating the underlying malignancy as well as immunological treatment such as immunosuppressants and intravenous immunoglobulins [4]. Prognosis of PLE varies according to the type of antibodies detected, it is usually less responsive to therapy and leads to serious outcomes with the presence of antibodies to intracellular antigens compared to antibodies to cell surface antigen which has higher response to immunotherapy [5]. Here, we present a case of PLE in a young male with thymoma, underscoring the importance of early recognition and multidisciplinary management.

## Case presentation

**Figure 1:**
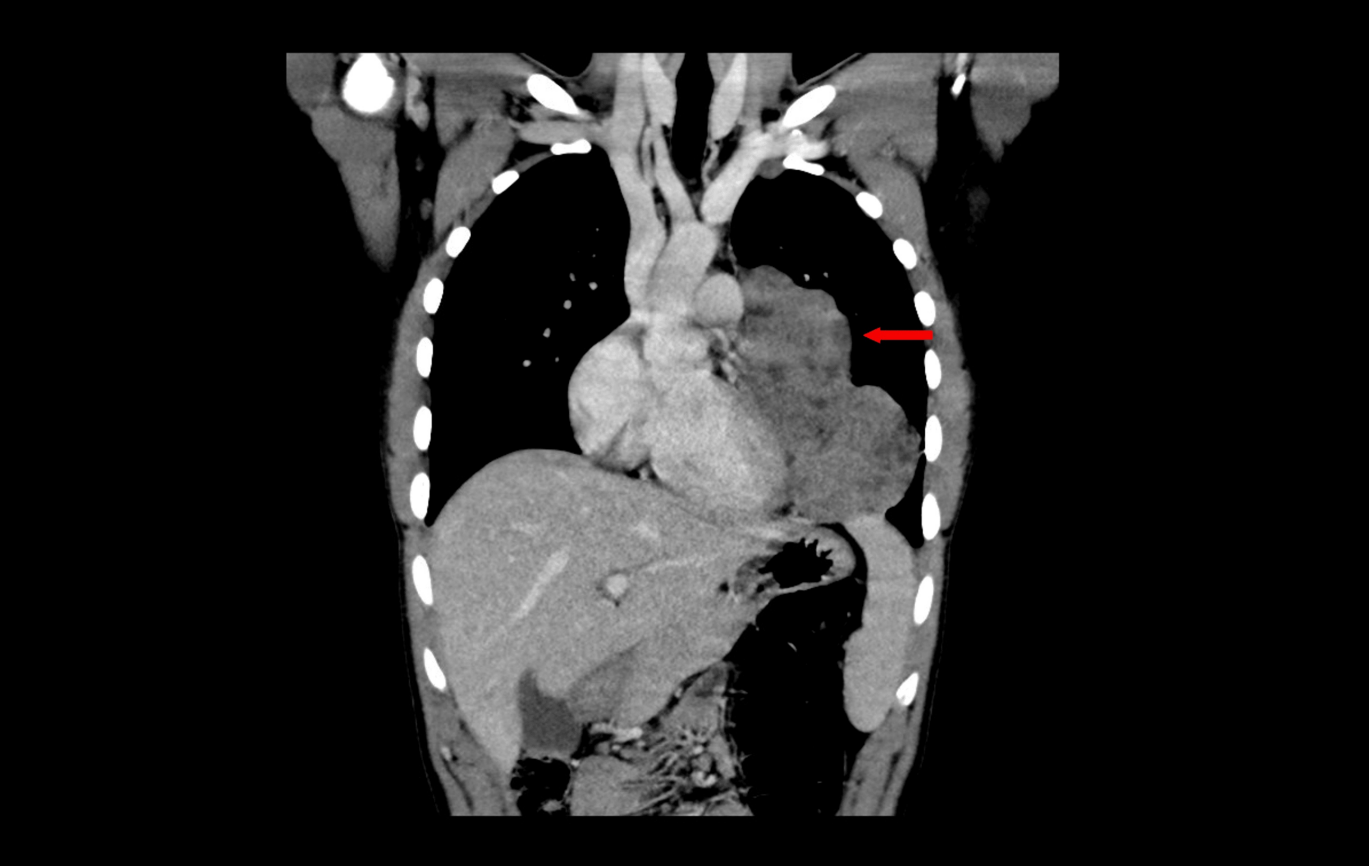
Coronal view of a computed tomography chest with intravenous contrast showing a large anterior mediastinal mass (arrow) draping over the left heart and pericardium extending along the mediastinal and posterior lateral chest wall pleura and lower chest into the left hemithorax.

**Figure 2:**
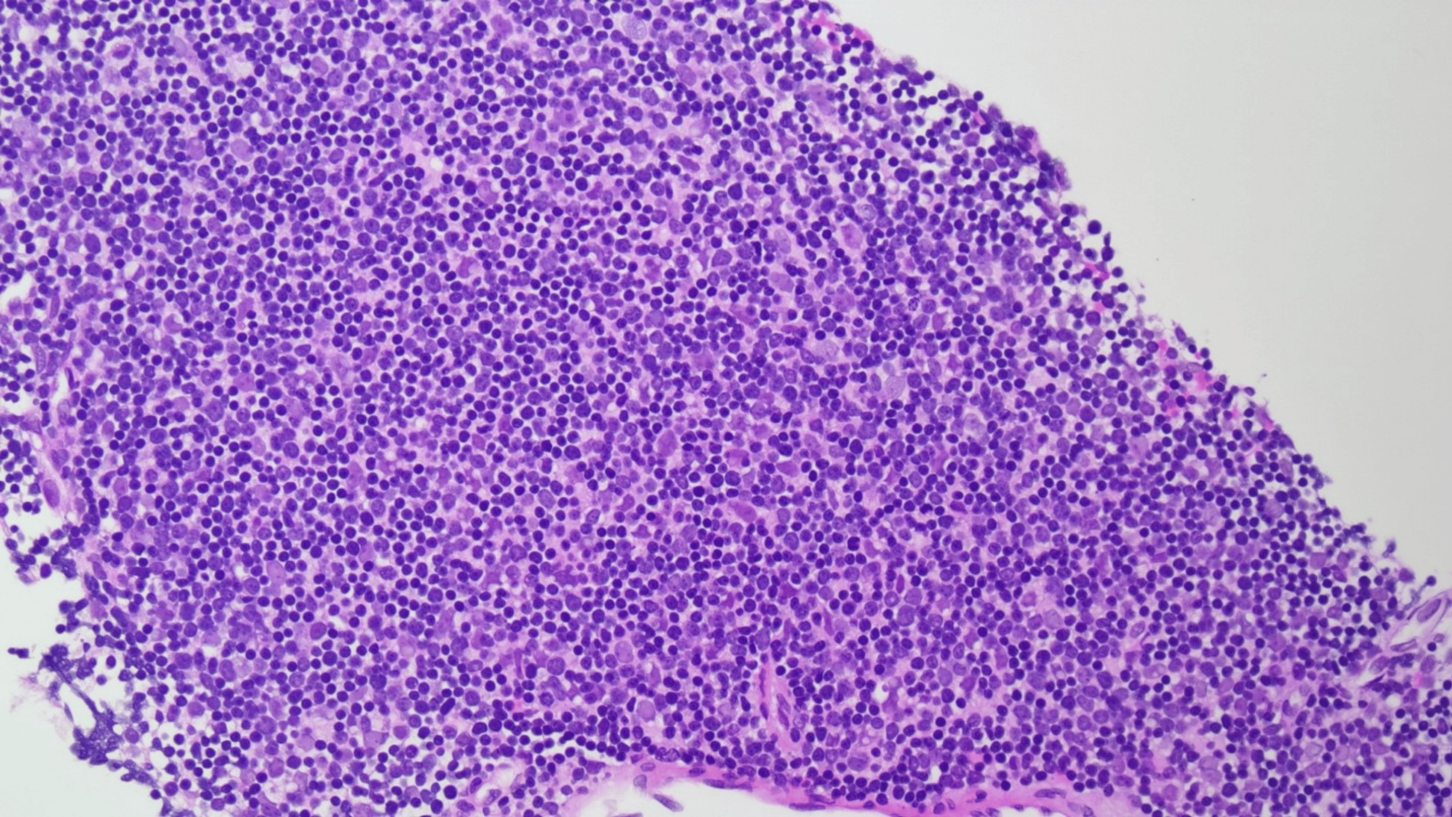
Photomicrograph of a biopsy from the mediastinum showing blue areas with predominant lymphocytes (thymocytes) and scattered polygonal epithelial cells (Hematoxylin and eosin, x200 magnification).

**Figure 3:**
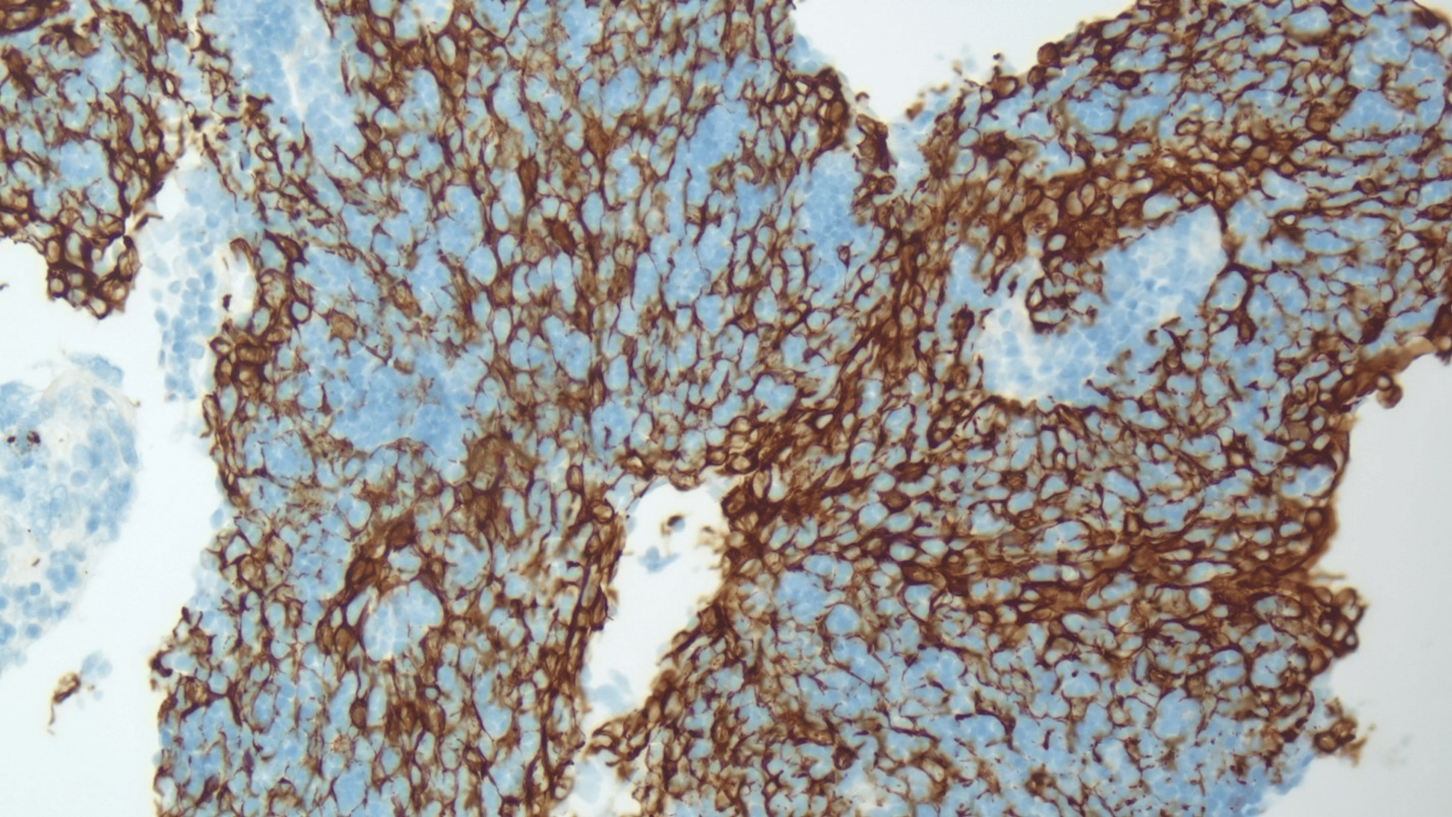
Microscopic image showing Keratin (AE1/AE3) immunostaining highlighting a diffuse meshwork of neoplastic cells (x200 magnification).

**Figure 4:**
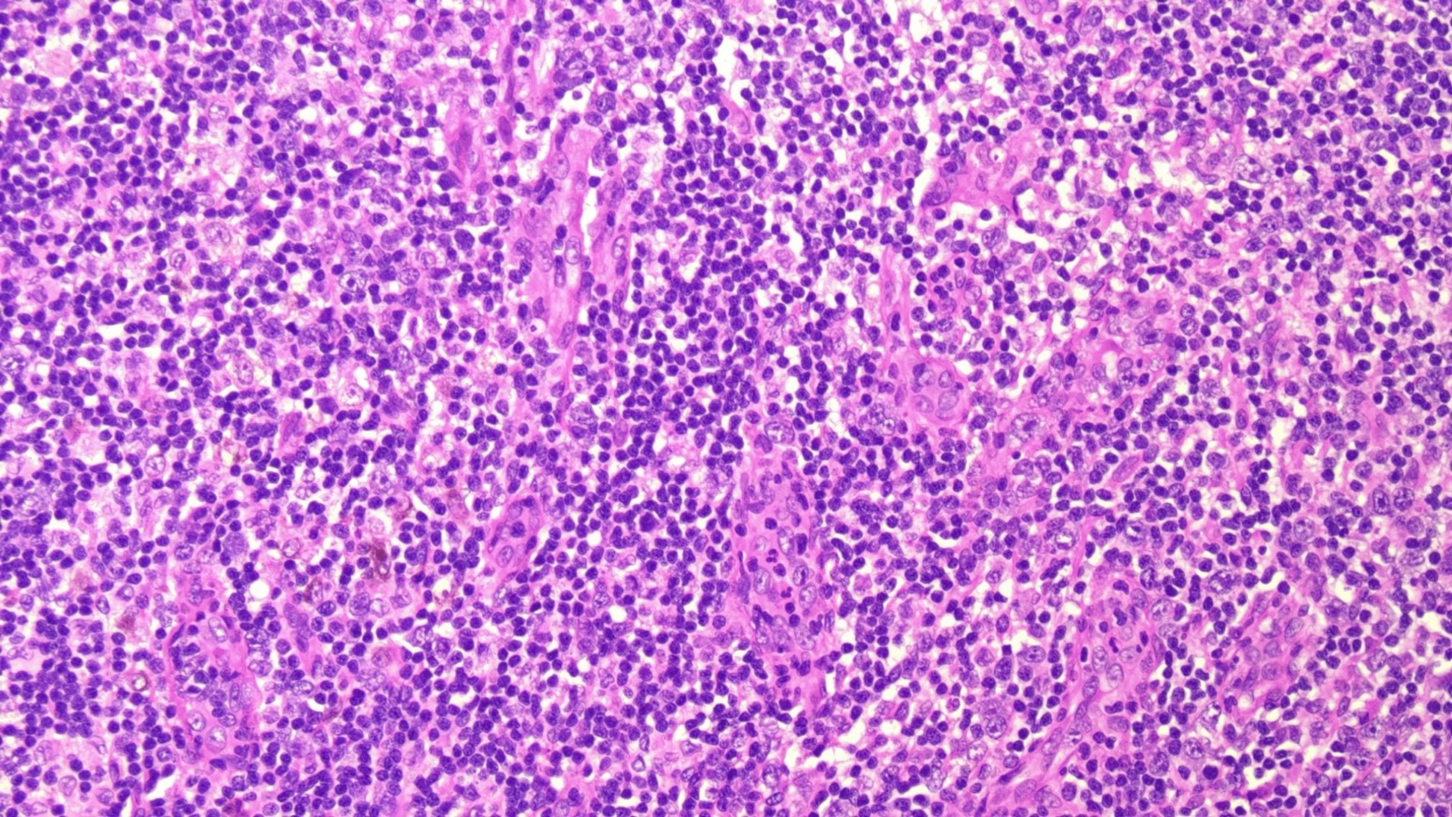
Photomicrograph of an excisional biopsy from the lymph node showing the interfollicular area with prominent vascular proliferation, mixed small lymphocytes, and plasma cells (Hematoxylin and eosin, x200 magnification).

A 17-year-old male with no significant medical history presented to the emergency department with a two-week history of chest pain and fever. He had no neurological symptoms. A chest X-ray revealed a lobulated mediastinal mass. A chest computed tomography (CT) revealed a large, enhancing anterior mediastinal mass extending along the pulmonary trunk and left hilum, draping over the pericardium and mediastinal pleura, causing mass effect on the major vessels and left heart border (Figure 1). Bilateral axillary lymphadenopathy was also noted. CT of the abdomen and pelvis demonstrated prominent bilateral inguinal and para-aortic lymphadenopathy, along with splenomegaly measuring 12.6 cm. Fluorodeoxyglucose positron emission tomography (FDG PET) revealed pleural disease involvement over the left lower lung lobe. Core biopsy of the mediastinal mass confirmed thymic tissue, characterized by a lymphocyte-rich background and large polygonal epithelial cells (Figure 2). Immunohistochemical staining confirmed epithelial cells positive for Pan-CK (Figure 3), p63, and CK5/6, while T-cells stained positive for CD99 and TdT. These findings were consistent with thymoma. Hematoxylin and eosin (H&E) staining of a formalin-fixed, paraffin-embedded tissue specimen from an excisional biopsy of the right inguinal lymph node revealed reactive follicles with interfollicular vascular and polytypic plasma cell proliferation (Figure 4). Immunohistochemical staining showed positivity for CD138, CD38, kappa, and lambda, and negative for human herpesvirus-8 (HHV-8), supporting a diagnosis of plasma cell-type idiopathic multicentric Castleman disease (iMCD). Human immunodeficiency virus (HIV) test was negative.

Due to the extent of pleural involvement and compression of major vessels and the heart border, surgical resection was deemed infeasible after a multidisciplinary team meeting, prompting initiation of systemic therapy. The patient received six cycles of systemic chemotherapy (CAP) comprising cyclophosphamide (500 mg/m²), doxorubicin (50 mg/m²), and cisplatin (50 mg/m²) every four weeks. The patient experienced significant clinical improvement following systemic therapy. Imaging revealed no significant interval size reduction of the mediastinal mass; however, FDG PET showed decreased metabolic activity. The multidisciplinary team decision was for watchful waiting and radiation therapy if patient becomes symptomatic or if he has evidence of progression.

Six months after completing systemic therapy, the patient presented with right-sided rhythmic facial twitching with intact awareness, not associated with involuntary arm movements, severe lumbar pain requiring intensified analgesic regimens, personality changes, and emotional lability. He also complained of excessive sweating and had an unexplained sinus tachycardia despite adequate analgesia. Magnetic resonance imaging (MRI) of the spine was unremarkable. A routine electroencephalogram was normal. MRI Brain with gadolinium was technically difficult due to significant susceptibility artifact due to dental braces and motion artifacts and it failed to show any abnormalities. Given a suspicion of paraneoplastic neurological disorder, serum and CSF were evaluated, including an auto-antibody panel. CSF examination revealed normal cell count, normal glucose and protein level, negative bacterial culture and herpes simplex virus polymerase chain reaction (Table 1). Auto-immune serology of both serum and CSF detected presence of high titers of antibodies against contactin-associated protein-like 2 (CASPR2) and leucine-rich glioma-inactivated 1 (LGI1). Other antibodies that were negative in the CSF include glutamate decarboxylase (GAD), dipeptidyl-peptidase-like protein 6 (DPPX), γ-aminobutyric acid (GABA) type B receptor, alpha-amino-3-hydroxy-5-methyl-4isoxazolepropionic acid receptors (AMPAR), glutamate receptor, amphiphysin, aquaporin 4, Ri, Ma and Ta antibodies. Dexamethasone 4 mg IV twice daily was started for 21 days. Two weeks later, intravenous immunoglobulin 2 grams per kilogram was administered daily over five days. The patient’s symptoms showed minimal response to steroids and immunoglobulin therapy. Subsequently, he received radiotherapy targeting the primary mass (20 Gray in five daily fractions), which led to significant and sustained improvement of his neurological symptoms at the time of this report.

**Table 1:** Cerebrospinal fluid examination CSF: Cerebrospinal Fluid, HSV PCR: Herpes Simplex Virus Polymerase Chain Reaction

CSF	Results	Normal values
White blood cell count	2 cells/mm	< 5 cells/mm
Glucose	6 mmol/L	> 2.2 mmol/L
Protein	0.262 g/L	0.15 - 0.4 g/L
Bacterial Culture	Negative	Negative
HSV PCR	Negative	Negative

## Discussion

PLE is most commonly associated with SCLC. A case series reported that 50% of patients diagnosed with limbic encephalitis had underlying SCLC [6]. A prospective study of SCLC patients reported a PLE prevalence of 1.5% [7].

Thymoma is frequently associated with paraneoplastic neurological syndromes, chiefly myasthenia gravis, as well as other non-neurological autoimmune syndromes [8]. Less commonly recognized paraneoplastic autoimmune neurological syndromes associated with thymoma include PLE and neuromyotonia. The association between PLE and thymoma was first reported in 1988 [9]. Since then, increased recognition of the paraneoplastic syndrome has led to the publication of numerous similar cases [10]. The characterization of the autoantibody associated with PLE in thymoma developed gradually [11]. One of the onconeural antibodies that was found to be frequent among patients with PLE and thymoma was the antibody directed against brain tissue-derived voltage gated potassium channel (VGKC). Further studies revealed these antibodies target two distinct antigens crucial for VGKC localization: CASPR2 and LGI1 [12]. Antibodies targeting CASPR2 and LGI1 have been associated with limbic encephalitis and neuromyotonia; the latter is characterized by peripheral nerve hyperexcitability [13]. In a series of 38 patients with CASPR2 auto-antibodies, the common symptoms included cognitive disturbances (79%), pain (61%), peripheral nerve hyperexcitability (54%), autonomic dysfunction (44%), and cerebellar dysfunction (35%) [14]. In a retrospective series of 43 patients with thymoma and autoimmune encephalitis, only seven patients had either CASPR2 or LGI1 antibodies, and only two patients had co-occurrence of both antibodies. More common antibodies found were GABA type A receptor and AMPAR. Concurrent positivity for antibodies against intracellular antigens such as collapsin response mediator protein 5 (CRMP5) was associated with poor response to therapy [15]. 

The case we report shares several findings with previously published case reports and series. The absence of pleocytosis and elevated protein levels in the CSF aligns with findings from multiple cases, including the first published report linking PLE to thymoma [9]. In one case series of 94 patients with autoimmune encephalitis [6], 26 patients had normal CSF examination. In another series of patients with CASPR2 autoimmune encephalitis, 65% of the patients had normal CSF examination [14]. The lack of limbic involvement on MRI is not uncommon, as less than half of PLE patients did not have evidence of limbic involvement on MRI in a large multinational database [16]. Regarding response to immunomodulatory agents, a cohort of lung cancer patients with PLE reported limited response to such treatment [17]. However, given the poor prognosis of the condition, immunomodulatory therapies in tandem with oncological therapies remain the cornerstone of PLE management.

A notable feature of this case is the co-occurrence of iMCD. While iMCD has been reported in association with various autoimmune disorders, its link to autoimmune encephalitis is not well established. Our literature review identified a single case report describing a 47-year-old man with the plasma cell variant iMCD who developed temporal lobe seizures and tested positive for voltage-gated potassium channel (VGKC) antibodies. [18]

The use of immune checkpoint inhibitors (ICIs) has grown exponentially and is now an integral part of the standard of care for various malignancies across different stages and treatment objectives. However, ICIs can activate autoreactive T cells, leading to immune-related adverse events (irAEs). Neurological irAEs are uncommon, corresponding to less than 5% of all irAEs, but are associated with high fatality rates [19]. A cohort of patients with ICI-related encephalitis that included patients with limbic encephalitis showed that it has poor response to immunosuppressants and carried overall poor prognosis [20]. As ICIs may trigger or worsen pre-existing autoimmune neurological syndromes, heightened vigilance in recognizing and managing it is crucial in patients receiving ICIs.

## Conclusions

In conclusion, this case report underscores several important points. The clinical presentation of PLE is often variable and requires high degree of clinical suspicion, a normal CSF examination and the absence of abnormalities on a brain MRI are not uncommon. Furthermore, when PLE is diagnosed in the setting of known malignancy, treating the underlying cancer can lead to improvement even if immunological treatments did not illicit response. The rising use of immune checkpoint inhibitors in oncology further underscores the importance of identifying and managing these syndromes promptly.
